# The Molecular Classification of Pheochromocytomas and Paragangliomas: Discovering the Genomic and Immune Landscape of Metastatic Disease

**DOI:** 10.1007/s12022-024-09830-3

**Published:** 2024-10-28

**Authors:** Carolijn J. M. de Bresser, Ronald R. de Krijger

**Affiliations:** 1 Department of Vascular Surgery, University Medical Center Utrecht, Heidelberglaan 100, 3584 CX Utrecht, The Netherlands; 2 Department of Pathology, University Medical Center Utrecht, Utrecht, The Netherlands; 3 Princess Máxima Center for Pediatric Oncology, Heidelberglaan 25, 3584 CS Utrecht, The Netherlands

**Keywords:** Pheochromocytoma, Adrenal paraganglioma, Extra-adrenal paraganglioma, Molecular classification, Immunohistochemistry, Biomarkers

## Abstract

Pheochromocytomas (PCCs) and paragangliomas (PGLs, together PPGLs) are the most hereditary tumors known. PPGLs were considered benign, but the fourth edition of the World Health Organisation (WHO) classification redefined all PPGLs as malignant neoplasms with variable metastatic potential. The metastatic rate differs based on histopathology, genetic background, size, and location of the tumor. The challenge in predicting metastatic disease lies in the absence of a clear genotype–phenotype correlation among the more than 20 identified genetic driver variants. Recent advances in molecular clustering based on underlying genetic alterations have paved the way for improved cluster-specific personalized treatments. However, despite some clusters demonstrating a higher propensity for metastatic disease, cluster-specific therapies have not yet been widely adopted in clinical practice. Comprehensive genomic profiling and transcriptomic analyses of large PPGL cohorts have identified potential new biomarkers that may influence metastatic potential. It appears that no single biomarker alone can reliably predict metastatic risk; instead, a combination of these biomarkers may be necessary to develop an effective prediction model for metastatic disease. This review evaluates current guidelines and recent genomic and transcriptomic findings, with the aim of accurately identifying novel biomarkers that could contribute to a predictive model for mPPGLs, thereby enhancing patient care and outcomes.

## Introduction

Pheochromocytomas (PCCs) and paragangliomas (PGLs) are rare neuroendocrine tumors originating from the embryologic neural crest and arise from clusters of paraganglia, dispersed from skull base to pelvic floor [1, 2]. Paraganglia originate from three major contributors: the adrenal medulla, hence the name adrenal PGL / pheochromocytoma, the sympathetic paraganglia, and parasympathetic paraganglia. The 2022 overview of the World Health Organization (WHO) on the classification of pheochromocytoma and paraganglioma (together: PPGLs) reclassified PCCs as adrenal PGLs, therefore categorizing this tumor as a PGL, collectively named PPGL [3].

Sympathetic paraganglia predominantly reside along the sympathetic chain, adrenal medulla, urinary bladder, organ of Zuckerkandl, and more rare localizations such as the posterior mediastinum, retroperitoneum, preaortic, suprarenal, paravertebral, and along the inferior hypogastric plexus [4, 5]. Parasympathetic paraganglia are predominantly located in the anterior thoracic region and in the head and neck area [1, 4]. Due to their widespread distribution, PGLs can develop in nearly any location in the body, except intraosseously. Although paragangliomas have been reported in the extremities, such occurrences are exceptionally rare [5]. The neuroectodermal origin of PGLs, especially sympathetic PGLs, enables them to hypersecrete catecholamines. Uncontrolled hypersecretion leads to sympathetic symptoms such as untreatable hypertension, paroxysmal tachycardia, anxiety, and excessive heavy sweating [6, 7]. Parasympathetic PGLs tend to be clinically nonfunctional in terms of catecholamine hypersecretion.

PGLs are hypervascular lesions that generally demonstrate slow growth [8]. The clinical presentation varies depending on the tumor’s location and neuroendocrine origin. Since the majority of PPGLs have a symptomatic nature, the most frequent symptoms are those associated with an excess of catecholamines, as previously described. Diagnosis of PPGLs usually depends on biochemical evidence of catecholamine overproduction by the tumor. Initial biochemical testing for PPGLs should include measurement of metanephrine (MN), normetanephrine (NM), and 3-methoxytyramine (3-MT) [9].

The variety in symptoms can be related to the differences in secretion of epinephrine and norepinephrine [10]. Distinct catecholamine biochemical phenotypic features of PPGLs depend on affected signaling pathways. Parasympathetic PGLs predominantly produce dopamine, due to lack of dopamine β-hydroxylase-mediated conversion (DBH) of dopamine to noradrenaline. Within chromaffin cells adrenaline is converted to metanephrine and noradrenalin into normetanephrine by catechol-O-methyltransferase (COMT), whereas in sympathetic nerves, in the absence of COMT, noradrenaline is deaminated by monoamine oxidase to dihydroxyphenylglycol (DHPG) [11]. In tumors that fail to (incompletely) convert dopamine to noradrenaline, dopamine can be O-methylated to 3-methoxytyramine [12]. In conclusion, adrenergic tumors are characterized by an elevation in plasma metanephrine levels, accounting for more than 5% of the combined increase of both normetanephrine and metanephrine. Conversely, noradrenergic tumors are identified by an elevated plasma normetanephrine level, with metanephrine levels contributing less than 5% that of both normetanephrine and metanephrine. Within the subset of noradrenergic tumors, dopaminergic tumors can be further distinguished by elevated levels of methoxytyramine [11, 12].

Radiologic work up for diagnostic anatomical imaging includes computed tomography (CT) or magnetic resonance imaging (MRI). Functional imaging with iodine-123 metaiodobenzylguanidine (MIBG), fluor-18-fludeoxyglucose (FDG), fluoro-18-dihydroxyphenylalanine (FDOPA), or gallium-68-dotaphenyltyrosineoctreotide (DOTATOC) is used for discrimination between paragangliomas and other lesions [13–16]. Tumor biopsy is not recommended in the diagnostic evaluation of these tumors due to the risk of hypertensive crisis and elevated risk of bleeding, given the tumor’s high degree of vascularization [17]. Treatment options differ, including wait-and-scan, surgical resection, radiotherapy, embolization, or a combination of these. The wait-and-scan approach is often considered for non-secreting head and neck paragangliomas. Targeted radionuclide therapies using iodine-131-metaiodobenzylguanidine (targeting the norepinephrine transporters) and peptide receptor radionuclide therapy (somatostatin analogues radiolabeled with either Lutetium-177 or Yttrium-90) have been presented as promising therapeutic options in the management of metastatic or inoperable PGLs [18].

Historically, PGLs were considered benign. However, the fourth edition of the WHO classification redefined all PCCs and PGLs as malignant neoplasms with variable metastatic potential. Recent estimations suggest that 10–15% of PCCs and up to 50% of abdominal paragangliomas will become metastatic [19, 20]. The metastatic rate differs on the basis of histopathology, genetic background, size, and location of the tumor [21, 22]. Distinction between metastasis and a second primary tumor is often challenging due to the widespread distribution of PPGLs throughout the body, including intraparenchymal locations in the brain, liver, and lungs. Therefore, metastases are defined as PPGLs occurring in areas where PPGLs are not normally present, such as lymph nodes or bone [3]. Five-year survival upon metastatic progression is heterogeneous and varies from 40 to 77% [23]. The malignant form of the disease remains challenging to predict and treat, as there are no established histopathological criteria to assess the risk of progression nor are there effective treatments available [24].

### Histopathology

The typical histological feature of PPGLs is the characteristic Zellballen pattern. This pattern is characterized by nests of tumor cells surrounded by non-tumorous sustentacular cells and a dense vascular network surrounding these nodules [25]. Nodule size has been suggested as an adverse histological criterion, related to increased risk of recurrence or metastasis and included in the multifactorial histological scoring systems, mentioned below. Similarly, a large number of histological characteristics, as well as some clinical, biochemical, or immunohistochemical criteria, have been related to potential worse outcome. Such criteria and characteristics have been grouped into several multifactorial prediction systems, including the Pheochromocytoma of the Adrenal gland Scaled Score (PASS), the Grading of Adrenal Pheochromocytoma and Paraganglioma (GAPP), and the COmposite Pheochromocytoma / Paraganglioma prognostic Score (COPPS) [26–28]. Although these prediction systems have their advantages, they generally have limited positive predictive value for clinical behavior and outcomes in PPGL patients. Consequently, they are not applicable to all PPGL types and are not currently endorsed by the WHO [3].

**Fig. 1:**
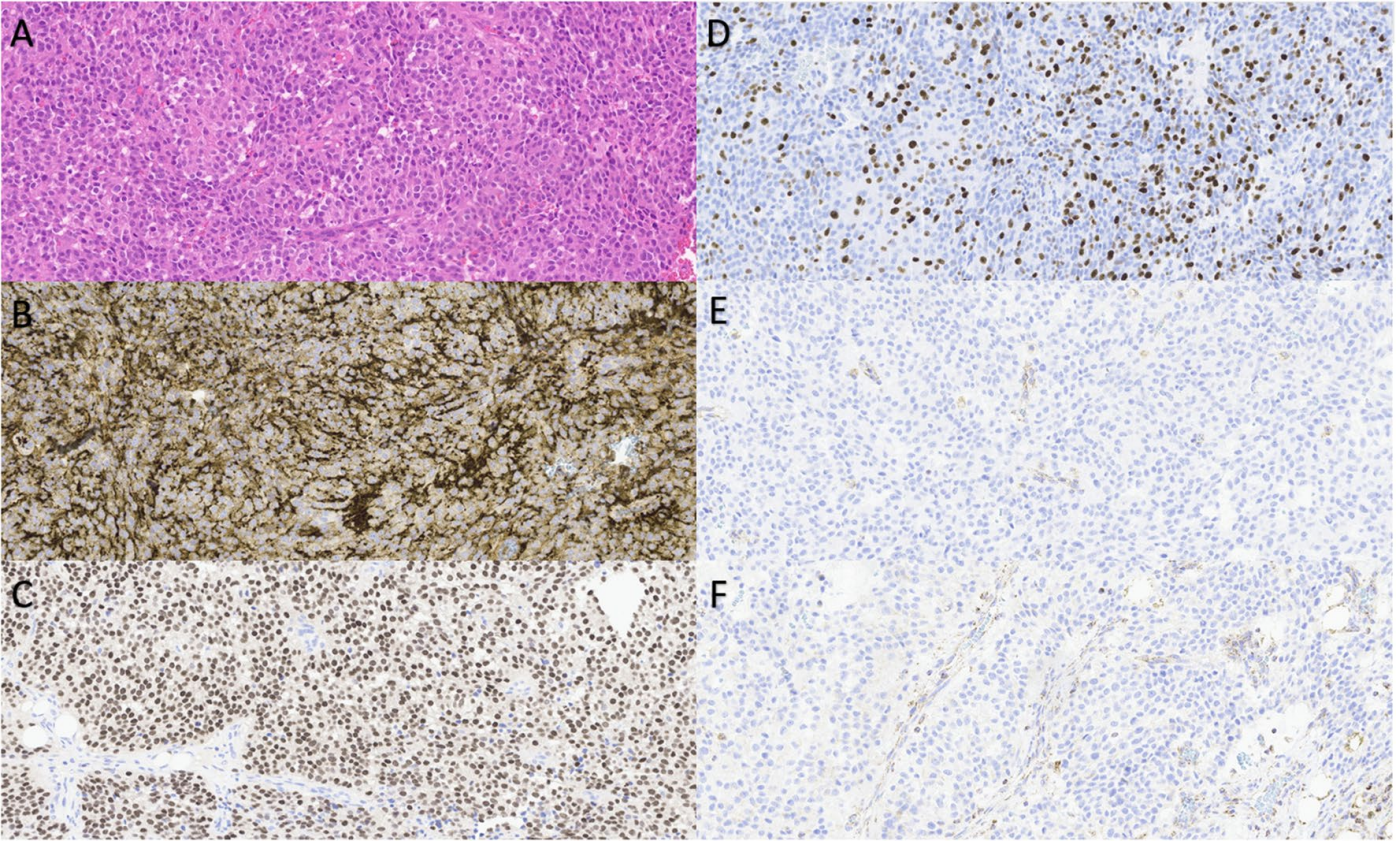
Morphological aspects of clinically aggressive pheochromocytoma. This clinically and morphologically aggressive pheochromocytoma presented in the left adrenal gland of a 17-year-old girl. The tumor shows cellular aspect with diffuse growth pattern and numerous mitoses (**A**), vascular invasion and ingrowth in the surrounding fat (not shown). The diagnosis was supported by strong staining for chromogranin-A (**B**), synaptophysin (not shown), and GATA3 (**C**). Keratin staining was lacking. Ki67 labeling index was high, up to 50% (**D**). Additional immunohistochemistry for succinate dehydrogenase subunit A (SDHA) and subunit B (SDHB) showed loss of expression of both proteins (**E**, **F**), suggesting a mutation in one of the SDH genes, highly likely in SDHA, underlining the role of morphology and immunohistochemistry in the diagnostic and prognostic approach of PPGL

While conventional PPGLs can often easily be recognized, especially by experienced pathologists, ancillary studies may be required to confirm the tumor’s nature. Specific immunohistochemical markers have been used for PPGL diagnosis (Fig. 1). The proliferation marker Ki-67 is an important criterium in the COPPS. This biomarker is a valuable tool for evaluating tumor growth rates due to its expression throughout the entire cell cycle, except during the resting G0 phase [29]. However, its sensitivity is limited to approximately 50%, making it insufficient as standalone marker for reliable assessment [30, 31]. Various studies in malignant pheochromocytomas have demonstrated elevated levels of Ki-67 expression [32–34].

The most universally used antibodies against neuroendocrine markers are chromogranin A, synaptophysin, and insulinoma-associated protein 1 (INSM1). These markers are valuable in the distinction of neuro-endocrine neoplasms (NEN) versus non neuro-endocrine neoplasms [4, 30]. In addition, PPGLs are characterized by certain relatively specific markers. One of these is the transcription factor GATA-binding protein 3 (GATA3), which is a nuclear marker with high sensitivity [35–37]. However, it should be noted that GATA3 expression is not exclusive to these tumors. It can also be observed in a variety of endocrine and non-endocrine tumors, including pituitary gonadotroph tumors, thyroid stimulating hormone (TSH) producing tumors, and some parathyroid tumors [38–40]. Furthermore, positive immunostaining of enzymes expressed by PPGLs may further differentiate. PPGLs express enzymes involved in the previously described catecholamine biosynthesis such as tyrosine hydroxylase (TH), dopamine beta-hydroxylase (DBH), and phenylethanolamine N-methyltransferase (PMNT). Some parasympathetic paragangliomas of the head and neck express choline acetyltransferase (ChAT), involved in acetylcholine biosynthesis [3, 25, 41, 42]. Virtually all PPGLs lack expression of keratins. Routine use of immunohistochemical stains for keratins (e.g., CAM5.2 and AE1/AE3) can further help to differentiate between NETs and PPGLs [4].

### Genetic Background

First evidence of the familial character of PGLs was already stated by Chase et al. in 1933 [43]. PPGLs have a strong genetic basis, and approximately 40% of all PPGLs have a hereditary cause with at least 20 genes associated with their development [44, 45]. Recently, several studies have reported an even higher proportion of hereditary PPGL cases, with figures up to 70%, as a result of the detection of more pathogenic variants in whole exome sequencing (WES) [46]. Historically, multiple endocrine neoplasia type 2 (MEN2), von Hippel-Lindau disease (VHL), and neurofibromatosis type 1 (NF1) were the archetypical hereditary cancer syndromes legated to PPGLs, but in the first decade of this millennium, a group of genes from the succinate dehydrogenase (SDH) complex were discovered to be involved in the pathogenesis of PPGLs. This enzyme complex participates in the conversion of succinate to fumarate in the Krebs cycle and also plays an important role in the electron transport chain. Genetic variation in any of these five genes causes dissemblance of the mitochondrial complex and loss of the SDH enzymatic activity [47]. Particularly, subunits A, B, C, D, and AF2 were discovered as carrying a high predisposition to PPGLs and certain other tumor types such as gastrointestinal stromal tumors (GIST), pituitary neuroendocrine tumors (PitNETs), and renal cell carcinomas (RCC) [47–50]. Autosomal dominant variants in any of these five genes are responsible for approximately 50% of all genetic variants and are expressed in five distinct genetic syndromes (Table 1). These familial paraganglioma syndromes are also referred to as succinate dehydrogenase deficient syndromes [51]. These patients often present with multifocal, early-onset tumors and a family history with PPGLs. Additionally, these patients may develop other tumor types associated with these syndromes. More rarely, germline variants are identified in transmembrane protein 127 (*TMEM 127*), malate dehydrogenase 2 (*MDH2*), fumarate hydratase (*FH*), and MYC-associated factor X (*MAX*) [5, 44].

**Table 1:** Clinical characteristics of SDHx-related syndromes [47, 52–54]

Gene	Syndrome	Transmission	Multiple lesions	Penetrance	Metastatic risk	Other manifestations
SDHD	Familial PGL type 1	AD, paternal	Yes	High	15–29%	GIST, PitNET, RCC
SDHAF2	Familial PGL type 2	AD	Yes	High	Not known	PitNET
SDHC	Familial PGL type 3	AD	Rare	Low	Low	GIST, RCC
SDHB	Familial PGL type 4	AD	Rare	Intermediate	35–75%	GIST, PitNET, RCC
SDHA	Familial PGL type 5	AD	Rare	Very low	30–66%	GIST, PitNET

*AD* autosomal dominant, *GIST* gastrointestinal stromal tumor, *PitNET* pituitary neuroendocrine tumor, *PGL* paraganglioma, *RCC* renal cell carcinoma, *SDHA* succinate dehydrogenase A gene, *SDHAF2* succinate dehydrogenase assembly factor 2, *SDHB* succinate dehydrogenase B gene, *SDHC* succinate dehydrogenase C gene, *SDHD* succinate dehydrogenase D gene

The mechanisms of somatic genetic alterations leading to tumorigenesis or malignant transformation are not fully understood. Somatic profiling has identified variants at varying frequencies in several genes, including SDHC, hypoxia-inducible factor 2-alpha (*HIF2α*, also known as endothelial PAS domain-containing protein 1 (*EPAS1*)), rearranged during transfection (*RET*), *VHL*, rat sarcoma (*RAS*), and *NF1* [55–63]. Recent studies reported that mechanisms of immortalization, frequently observed in carcinomas, involving telomere deregulation are implicated in the metastatic progression of PPGLs [64]. Somatic variants in genes such as alpha-thalassemia mental retardation X-linked (*ATRX*) [65], tumor suppressor protein 53 (*TP53*), lysine methyltransferase 2D (*KMT2D*) [66], set domain containing 2 (*SETD2*), and telomerase reverse transcriptase (*TERT*) promotor cause either telomerase upregulation or alternative lengthening of telomeres [67, 68]. Notably, *ATRX* genetic variants are associated with alternative lengthening of telomeres in metastatic PPGLs, sometimes in combination with nucleolar protein 10 (*NOP10*) [65, 69]. Furthermore, re-expression of telomerases, characterized by elevated *TERT* expression, is also detected in metastatic PPGLs [70, 71]. Activation of the telomerase gene *TERT* and *ATRX* loss of function variants have been associated with poor prognosis in PPGL [67, 71, 72].

However, there are still some patients with clinical indicators of hereditary disease, which are not explained by variants in these genes. Genetic testing via WES of all patients with PPGLs has been recommended, due to this high heritability rate [9]. It is anticipated that further detailed genetic testing, such as through whole genome sequencing, may either uncover molecular variants in hitherto poorly analyzed parts of genes, including promoter or enhancer regions, or in currently unknown predisposition genes, most likely related to one of the pathways described below. The many genes involved in PPGLs seem to be converging into three pathogenic pathways causing tumorigenesis: the pseudohypoxia pathway, the kinase pathway, and the Wnt-altered pathway. This review will provide an overview of these pathways and include DNA and transcriptome-related analyses for the classification of PPGL biomarkers, which may contribute in the development of future metastatic risk prediction models.

## Molecular Pathogenesis of PGL

**Fig. 2:**
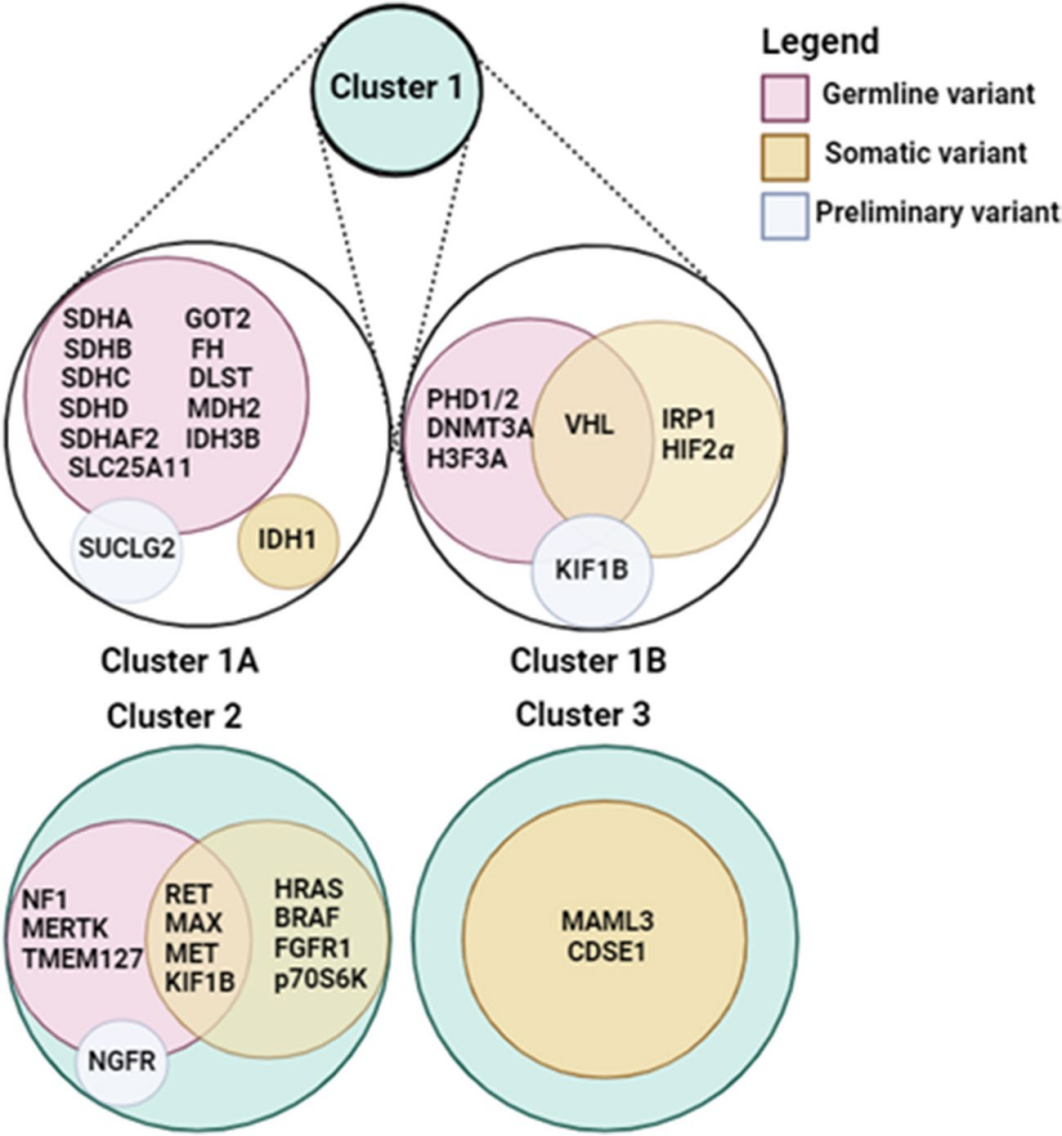
Cluster specific genetic variants. Genetic variants affecting cluster 1A, cluster 1B, cluster 2, and cluster 3 are illustrated. These variants are categorized into three groups: germline variants (red), somatic variants (orange), and preliminary variants (light blue). Overlapping regions represent variants classified as both germline and somatic. Preliminary variants are suggested to be causative of PPGLs, but require further validation through additional studies

A strong genotype–phenotype correlation in PPGLs is found with respect to the tumor’s catecholamine profile, anatomic location, and risk of metastatic spread. Functional, pathogenesis-based classification of PPGLs allows a better understanding of actual tumorigenesis, location, and malignant potential. The Cancer Genome Atlas (TCGA) proposed a division of PPGLs into three main molecular subgroups, linked by distinct driver genes: pseudohypoxia pathway (Cluster 1: Krebs cycle related (1A) and non-Krebs cycle related (1B), *SDHA*, *SDHB*, *SDHC*, *SDHD*, *SDHAF2*, *FH*, *VHL*, *HIF2α*, and *EGLN1*)); kinase signaling (Cluster 2: *RET*, *NF1*, *TMEM127*, *MAX*, Harvey Rat Sarcoma Viral Oncoprotein (*HRAS*), Fibroblast Growth Factor Receptor 1 (*FGFR1*), and MET Proto-Oncogene, Receptor Tyrosine Kinase (*MET*)); and Wnt-altered (mastermind-like transcriptional coactivator 3 (*MAML3*) or Cold Shock Domain Containing E1 (*CSDE1*)) [24, 72, 73] (Fig. 2).

### Cluster 1A: Pseudohypoxia PPGLs–Tricarboxylic Acid Cycle (TCA) Aberrant

TCA cycle aberrant PPGLs exhibit the highest risk of metastatic dissemination [74]. Germline variants in SDH subunits A, B, C, and D, as well as the assembly factor (*SDHAF2*) and *FH*, activate the physiological response to hypoxia, even under normoxic conditions. Other notable variations include germline variants in glutamic-oxaloacetic transaminase 2 (*GOT2*), 2-oxoglutarate-malate carrier (*SLC25A11*), dihydrolipoamide S-succinyltransferase (*DLST*), *MDH2*, isocitrate dehydrogenase 3 (*IDH3B*), and a somatic alteration in isocitrate dehydrogenase 1 (*IDH1*) [1, 75–77].

These genetic variants result in inactivation of the tricarboxylic acid cycle, leading to the accumulation of extramitochondrial metabolic intermediates (oncometabolites) such as fumarate, succinate, and alpha-ketoglutarate derivatives and severe impairment of mitochondrial oxidative phosphorylation [74, 78, 79]. Accumulation of these oncometabolites as well as certain genetic variants (Cluster 1B: prolyl hydroxylase 1 and 2 (*PHD1 / 2*, *also known as Egl-9 Family Hypoxia Inducible factors 1 / 2* (*EGLN1/2*)), *VHL*) promotes DNA hypermethylation. Furthermore, one important group responsible for genomic demethylation is the group of ten-eleven translocation (TET) dioxygenases inhibitor enzymes. TET inhibition results in tumor development and metastatic spread [80, 81]. DNA hypermethylation inactivates tumor suppressor genes including *PHD1/2* and activates the hypoxia-inducible factor (HIF) oncogenic pathway by decreased HIF-α hydroxylation and lower HIF-α ubiquitination/degradation [4, 82]. Elevated levels of HIF-α induce expression of genes that facilitate angiogenesis, tumor extravasation, migration, invasion, and metastasis [68, 83].

Furthermore eight germline variants in the succinate-CoA ligase GDP-forming subunit beta (*SUCLG2*) have been identified in apparently sporadic PPGL patients [84]. This subunit has a role in the TCA cycle by coupling the succinyl-CoA and guanosine triphosphate (GTP) synthesis, thereby mediating the level of phosphorylation [85]. These germline variants suggest a potential role of *SUCLG2* as a PPGL-associated candidate gene [84].

### Cluster 1B: Pseudohypoxia PPGLs–Hypoxia-Signaling Pathway/TCA Cycle Non-aberrant

Variants in signaling pathways that regulate HIF-mediated transcription are not primarily associated to the Krebs cycle and therefore form a separate group. Germline variants in the hypoxia-related genes such as *VHL* and *PHD1/2* and somatic mutations including VHL, *HIF2α*, and iron regulatory protein 1 (*IRP1*) lead to a decreased degradation of HIF, resulting in increased HIF signaling and the promotion of angiogenesis and proliferation [1, 86, 87]. Due to normal epigenetic regulation, with no inhibition of TET-enzymes, this group is less prone to metastatic spread compared to the TCA-aberrant group.

In addition, germline variants in DNA methyltransferase (*DNMT3A*) and postzygotic histone subunit gene (*H3F3A*) are directly involved in DNA hypermethylation [57, 88]. Previously, germline and somatic variants in kinesin family member 1B (*KIF1B*) have been reported as a contributor to hypermethylation as well [89, 90]. Although pathogenicity cannot be fully excluded, *KIF1B* is probably not a susceptibility gene. Since the initial report in 2008, there have been only few identified cases with a germline variant in *KIF1B* (*n* = 4, prevalence 0.7–1.3% in the individual studies), of which 3 were considered variants of unknown significance. In addition, in the initial case and the four other cases that was presumed pathogenic, somatic loss of heterozygosity was not found for the wildtype allele [90–93].

### Cluster 2: Kinase Pathway Aberrant

Cluster 2 alterations result in overactivation of the tyrosine kinase-linked signaling pathways. Approximately 50% of all PPGLs harbor a germline or somatic mutation in one of the associated genes. These tumors are predominantly PCCs with low metastatic potential. Different genetic variants affect different signaling pathways leading to sustained cell growth, survival, proliferation, and angiogenesis [24, 59, 72, 73].

The proto-oncogene *RET* encodes for a receptor tyrosine kinase activating phosphatidylinositol-3-kinase (PI3K)/AKT (formerly known as protein kinase B (PKB)/ mammalian target of rapamycin complex 1 [PI3K/mTORC1] and MAPK/ERK (or Ras-Raf-MEK-ERK) pathway [94–97]. Upon RET activation, uncontrolled stimulation of the signaling pathways downstream of RET accounts for uncontrolled mitogenesis, decreased protection from programmed cell death, and increased cellular motility [98].

The *NF1* tumor suppressor gene encodes the NF1-protein, which is Ras GTPase-activating (RasGAP) and thereby inactivating RAS. *NF1* variations have been associated with loss of inhibition, leading to the persistent activation of RAS and mTORC1, and subsequently contributing to the involvement of the PI3K/AKT pathway in tumorigenesis [99–101].

Tumors with a variant in *TMEM127* display increased mTORC1 signaling, thereby activating cell growth and proliferation [102, 103]. The *MAX* genes (germline and somatic) encode the MAX protein, working as a co-factor of the proto-oncogene MYC mediating its function as transcription factor by heterodimerization causing abnormal interaction between protein subunits [104, 105]. The significant interaction between the PI3K/AKT/mTORC1 and MAPK/ERK pathways has previously been reported [106, 107]. It is conceivable that other factors in these pathways are affected, such as somatic variants in ribosomal protein S6 kinase beta-1 (p70S6K), playing a crucial role in controlling cell cycle, growth, and survival [108]. Rare germline variants such as MER proto-oncogene, tyrosine kinase (*MERTK*) [57]; MET proto-oncogene, receptor tyrosine kinase (MET, germline and somatic) [57], and somatic alterations such as *KIF1B* (germline and somatic) [109], Harvey rat sarcoma viral oncoprotein (*HRAS*) [110], B-rapidly accelerated fibrosarcoma (*BRAF*) [110], nerve growth factor receptor (*NGFR*) [72], and fibroblast growth factor receptor 1 (*FGFR1*) have recently been added to this pathway [24, 57, 72, 111]. *HRAS* and *FGFR1* mutations are more common in the Chinese (*HRAS*, 16.5%; *FGFR1*, 9.8%) than European population (*HRAS*, 9.8%; *FGFR1*, 2.2%) [112]. *NGFR* is not yet supported by other studies.

### Cluster 3: Wingless Type (Wnt) Pathway

The Wnt signaling-related cluster primarily includes PPGLs driven by the somatic variants in mastermind-like transcriptional coactivator 3 (*MAML3*) and cold shock domain containing E1 (*CDSE1*) [72, 113]. MAML3 serves as an essential transcriptional coactivator for mediating cellular responses in the oncogenic NOTCH signaling pathway [113, 114]. Fusion of transcription factor 4 (*TCF4*) or upstream binding transcription factor (UBTF) promotor region with MAML3 can lead to its overexpression, which is associated with an increased growth rate of PPGLs. *CDSE1* plays a pivotal role in translation initiation, RNA stability, cell-type-specific apoptosis, differentiation, and neuronal development [115, 116]. PPGLs from cluster 3 contain an increased risk of metastatic dissemination.

It is conceivable, based on the many enzymes and other proteins involved in each of the abovementioned pathways, that further genetic variants will be uncovered in known or hitherto unknown genes that play a role in the tricarboxylic cycle, the pseudohypoxia pathway, or in the kinase signaling pathway. This is supported by the fact that there are still familial cases, which have not been attributed to currently known genes.

## Developments in the Mutational Landscape of Metastatic PPGLs

In 2017, the WHO introduced staging of PPGLs with the tumor, node, and metastasis (TNM) classification to classify the extent of cancer spread, established by The American Joint Committee on Cancer/Union for International Cancer Control (AJCC/UICC) [117]. While the harmonization of disease classification is beneficial, this staging system still has its limitations. Among PPGL patients within the same stage, the clinical outcome can be very different [118]. Furthermore, the TNM classification for PPGLs lacks adequate staging of non-functional PPGLs of any size and does not consider metastatic risk [117]. Some PPGL genotype–phenotype correlations have been identified, but disease management remains challenging due to variable clinical behavior, lack of accurate markers for metastatic risk, and insufficient diagnostic standards for metastatic staging [1, 17, 119]. The previously identified clusters categorize PPGLs into three distinct genomic subtypes. However, the studies that established these classifications included a limited number of metastatic cases [24, 72]. A recent study by Calsina et al. utilized WES and RNA sequencing (RNA-Seq) for genomic profiling, revealing new markers for metastatic risk estimation. The findings presented in this study suggest that incorporating immune parameters could be valuable in the clinical management of PPGLs for prognostication and at the same time identifying patients who might benefit from immunotherapy [119].

WES data confirmed a previously observed association between higher tumor mutational burden (TMB) and metastatic behavior [72, 120]. TMB is defined as the total number of nonsynonymous mutations per coding region of a tumor genome [121]. Variability of TMB has been observed across and within cancer types [122]. Lung and head and neck cancer show less variability in TMB, compared to colon, bladder, and uterine cancer [123]. An increase in TMB was observed in progression from non-metastatic primary tumors to primary metastatic PPGLs (mPPGLs) and metastases. Cluster 3 (Wnt-altered subtype) exhibited the highest TMB values, irrespective of tumor behavior [119].

Microsatellite instability (MSI) contributes to hypermutated phenotypes in various cancer types [124]. Clinical behavior of PPGLs is linked to increased MSI scores, which progressively rises from non-metastatic tumors to primary metastatic tumors and metastases. A significant positive correlation between MSI and TMB has been identified, and both are associated with an increased risk of metastasis, indicating that they may serve as independent predictors of metastatic potential. Additionally, higher TMB and MSI scores were also associated with a shorter time to metastatic progression (TTP). Metastatic PPGLs with *ATRX/TERT* variants exhibited higher TMB and MSI, higher Ki-67 labeling index, and larger tumor size, compared to *ATRX/TERT* non-altered samples.

WES data was also utilized to analyze somatic copy-number alteration (SCNA) profiles, observing an increase in SCNA events from non-metastatic PPGLs, to more aggressive, metastatic tumors and eventually metastases. Additionally, a trend of shortened TTP was seen with increasing SCNA events, and a higher SCNA burden was observed in *ATRX/TERT*-altered tumors. Four altered arm-level SCNAs were identified between metastatic and non-metastatic primary tumors. A gain of the entire chromosome 5 was associated with shorter TTP, consistent with the *TERT* location at 5p 15.33 [119].

RNA-Seq has been used to analyze the transcriptomic landscape of mPPGLs revealing a significant overrepresentation of genes associated with the cell cycle, G1/S-specific transcription, p53 signaling, and DNA damage response. Cyclin-dependent kinase 1 (*CDK1*) gene, enriched in previously mentioned 4 gene sets, exhibited the most significant association with metastatic risk when compared to both non-metastatic and metastatic disease [119].

Calsina et al. aimed to develop a metastatic risk classifier for PPGLs incorporating several potential biomarkers: high MSI score; high TMB; gain of chromosome 5 and high *CDK1* expression, in combination with the previously described marker germline variants of Cluster 1A; *MAML3*-fusion; *TERT-*alterations; *ATRX-mutation*; and high Ki-67 expression [26, 65, 72, 83, 125]. The best classifier took only four biomarkers into account, with a sensitivity of 100% to predict mPPGL: *ATRX*-mutations, high MSI score, high *CDK1* expression, and *MAML3*-fusions. While this study deserves great merit for its broad scope with regard to potential metastasis-related parameters, it should be noted that most of the proposed parameters have not been extensively investigated before in metastasized and non-metastasized PPGL. In addition, due to the rarity of PPGL in general and mPPGL in particular, numbers of cases in the current study are limited. Therefore, further validation of this tool, on a large independent cohort of PPGL patients, is required [119].

Various histological features have been reported to be associated with an increased risk of PPGL metastasis, including invasion of soft tissue and blood vessels, particular architectural patterns including hypercellularity and large confluent nests, comedo-type necrosis, and a high mitotic count or Ki-67 proliferative index [26, 27, 126–128]. These parameters primarily focus on tumor-cell characteristics, often overlooking intratumor heterogeneity, and the impact of the tumor microenvironment (TME), consisting of non-tumorous cells and various immune components [129]. Previous studies in colorectal cancer have demonstrated that type, density, and location of immune cells can provide prognostic information that is superior to, and independent of, TNM classification [130]. The TME, which evolves as tumors develop, is complex and consists of non-cancer cells such as endothelial cells, immune inflammatory cells, pericytes, fibroblasts, and a dynamically remodeled extracellular matrix [131, 132]. Correlations have been observed between tumor-associated macrophages and increased grade non-small cell lung carcinoma (NSCLC) patients [133]. Additionally, the TME has been studied across three major immune clusters associated with pediatric central nervous system tumors: medulloblastomas (MB), malignant rhabdoid tumors (MRT), and pediatric high-grade gliomas (pHGG). Findings indicate a relative increase of CD4 + T-cell infiltration in MBs, with a lesser extent observed in MRTs. Conversely, pHGGs and certain clusters of MRTs exhibit a relatively higher infiltration of monocytes [134]. Understanding and clustering the TME can enhance the precision of targeted therapies and immunotherapeutic strategies. Different clustering categories have been proposed recently. Bagaev et al. identified four TME subtypes that are consistent across various cancers and correlate with immunotherapy response in melanoma, bladder, and gastric cancers. The four molecular profiles are clustered into four distinct TMEs: immune-enriched; fibrotic (IE/F); immune-enriched; non-fibrotic (IE); fibrotic (F); and immune-depleted (D). In PPGLs, the immune-enriched non-fibrotic subtype was associated with an immune-active TME and exhibited the best prognosis. This group contained the highest proportion of non-mPPGLs [119]. Conversely, in this same immune-enriched non-fibrotic subtype, more aggressive cancers, predominantly melanoma, bladder, and gastric cancer, responded to immune checkpoint inhibitors [135]. Unfortunately, in the case of mPPGLs, the fibrotic subtype was most prevalent and was associated with the poorest response to immunotherapy [119, 135].

Another TME classification has been introduced by Thorsson et al. Six immune subtypes (C1-C6) were found based on immunogenomic analysis of over 10,000 tumors utilizing data by TCGA. These six categories comprise the following: C, wound healing; C2, IFN-γ dominant; C3, inflammatory; C4, lymphocyte depleted; C5, immunologically quiet; C6, TGF-β dominant. PPGLs are enriched in C4, displaying more prominent macrophages. These six immune subtypes were linked to prognostic outcomes, genetic variants, and immune modulatory changes, likely influencing the distinct observed immune environments. PPGLs subtype C4, together with C6, was correlated with shorter TTP and the worst prognosis. These subtypes displayed an immunosuppressed TME dominated by macrophages and low lymphocytic infiltrate, expected to have a poor outcome [136].

The composition of the TME plays a crucial role in shaping tumor-immune interactions and influencing treatment responses [137]. To optimize the efficacy of immunotherapy or its combination with targeted antigens, treatment strategies must be customized to address the specific characteristics of the TME [134]. The TME can be characterized and quantified using techniques such as immunohistochemistry (IHC), especially cyclic IHC, in which large numbers of targets can be simultaneously investigated by fluorescence techniques [138]. In addition, fluorescence-assisted cytometry (FACS), mass cytometry (Cy-TOF), and RNA-Seq may be used for this purpose as well.

Despite the advances described above, it appears that our knowledge on the TME is only beginning to emerge and that further detailed collaborative research, both specifically for PPGL, but taking pan-cancer approaches in consideration as well, will be needed to dissect relevant players and develop targeted immunotherapies.

While PPGLs have been incorporated into extensive genomic and transcriptomic studies, the lack of replication in PPGL-specific cohorts focused on metastatic disease has impeded the validation of these findings. As a result, the role and characteristics of the TME in PPGLs remain largely unexplored.

**Fig. 3:**
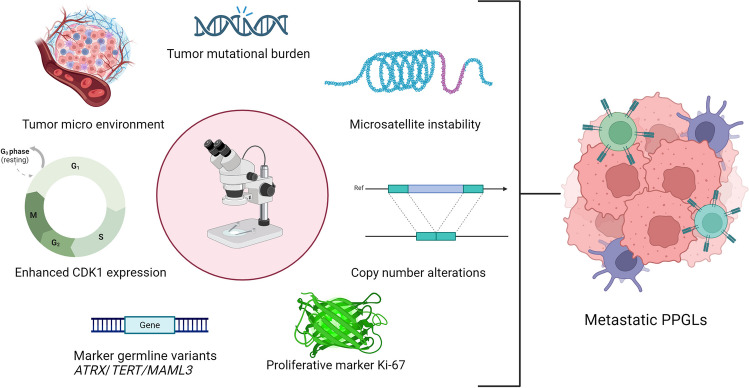
Overview of seven potential biomarkers contributing to the development of metastatic pheochromocytomas and paragangliomas. Histopathology serves as the foundation for assessing the potential metastatic risk of PPGLs, with evaluation of Ki-67 and the tumor micro environment (TME) relying on this. Molecular techniques, building on morphological analyses, are utilized to confirm the presence of potential biomarkers (tumor mutational burden (TMB); enhanced CDK1 expression, tumor indicative of metastatic risk in pathological samples)

To address this gap, it is crucial to conduct research involving large cohorts of both metastatic and non-metastatic PPGLs using these advanced methods to provide deeper insight into the TME of PPGLs and to assess the efficacy of current immune checkpoint inhibitors in targeting mPPGLs. Additionally, the seven possible biomarkers illustrated in Fig. 3 each contribute to the metastatic risk of PPGLs, forming a multifactorial predictive risk model to estimate the potential metastatic risk of PPGL patients. Further research is needed to validate these biomarkers and elucidate their role within the TME. Such efforts will enhance our ability to predict metastatic risk and optimize treatment strategies for PPGL patients.

## Conclusion

PPGLs are the most hereditary tumors we know, with estimates of heritability increasing from 40% to as high as 70% in more recent studies. The strong genetic predisposition necessitates routine screening and follow-up for patients and family members, which imposes a significant burden on both affected individuals and their healthy relatives. Given the indolent growth and the lack of consistent correlation between tumor genotype and phenotype, there remains a critical unmet need in managing these tumors, particularly regarding their unpredictable clinical behavior and potential metastatic risk.

The complexity of PPGL management often requires a multidisciplinary team, typically comprising of surgeons, endocrinologists, clinical geneticists, radiologists, pathologists, and nuclear physicians to collaborate and determine the optimal treatment for PPGL patients [139]. Management approaches for mPPGLs are currently influenced by factors such as biochemical phenotype, genetic variant (and underlying mutational landscape), tumor size, multifocality, and presence or absence of metastases. These factors often correlate with a higher risk of future metastasis [140–143].

Recent advances in understanding tumorigenesis of PPGLs have led to a significant progress in classifying these tumors into three molecular clusters based on distinct germline and somatic variants. Although these molecular clusters provide some insights into genotype–phenotype relationships and metastatic risk, direct correlations remain limited.

Genomic profiling and transcriptomic analysis have identified several potential biomarkers that may influence metastatic potential. However, it appears that a combination of these biomarkers, rather than any single one, is required to predict metastatic risk more accurately. Novel potential biomarkers include *ATRX*-mutations, high TMB, chromosome 5 gain, *TERT*-alterations, high Ki-67 expression, high MSI score, high *CDK1* expression, and *MAML3*-fusions. Understanding the TME in PPGLs may also improve the application of targeted immunotherapies for metastatic diseases, though this area of research is still rudimentary and requires further exploration.

International studies are essential to validate these biomarkers and develop a robust prediction model for metastatic disease. Such a model would enhance our ability to predict the clinical behavior of PPGLs, estimate the likelihood of metastasis, and ultimately improve patient outcomes.

## Data Availability

No datasets were generated or analysed during the current study.
